# Simulating Cor pulmonale in chronic obstructive pulmonary disease via cigarette smoke exposure and left pulmonary artery ligation in mice

**DOI:** 10.14814/phy2.70727

**Published:** 2026-01-09

**Authors:** Xilong Wang, Guanjin Chen, Qianwen Bai, Yuhang Huang, Kai Zhong, Zhuoji Ma, Shuiying Zhang, Neng Wang, Xin Chen, Tao Wang

**Affiliations:** ^1^ Department of Pulmonary and Critical Care Medicine, Zhujiang Hospital Southern Medical University Guangzhou China; ^2^ State Key Laboratory of Respiratory Diseases, Guangdong Key Laboratory of Vascular Diseases, Guangzhou Institute of Respiratory Health The First Affiliated Hospital of Guangzhou Medical University Guangzhou Guangdong China; ^3^ Department of Cardiology, Suizhou Hospital Hubei University of Medicine Suizhou Hubei China

**Keywords:** animal model, chronic obstructive pulmonary disease, Cor pulmonale, pulmonary hypertension, right heart failure

## Abstract

Cor pulmonale, indicative of right heart failure (RHF), is precipitated by pulmonary conditions that escalate pulmonary arterial pressure. This complication is notably prevalent in patients with chronic obstructive pulmonary disease (COPD), and is recognized as an independent predictor of adverse outcomes. Despite its significance, the lack of appropriate animal models has hindered the development of therapies for cor pulmonale in COPD patients. Therefore, we aimed to establish a mouse model that mimics the essential pathological features of COPD‐cor pulmonale. All mice underwent thoracic surgery, including left pulmonary artery ligation (LPAL) or sham surgery (artery exposed without ligation). Following a 2‐week recuperation, the mice were exposed to cigarette smoke or room air for 28 weeks. At the end of the exposure, pulmonary function, right ventricular hemodynamics, and histological alterations were determined. CD31, α‐SMA, and CD68 were detected by immunofluorescence and immunohistochemistry to show vascular and macrophage changes. Mice subjected to LPAL and cigarette smoke exposure exhibited COPD‐like features, including impaired lung function, emphysematous alterations, pulmonary inflammatory cell infiltration, and airway remodeling, accompanied by the increase in right ventricular systolic pressure, right ventricular hypertrophy, fibrosis, macrophage aggregation, and reduced capillary density. This model, integrating cigarette smoke exposure with LPAL, effectively replicates the critical pathological features of COPD‐cor pulmonale.

## Graphical abstract

Mice subjected to LPAL and cigarette smoke exposure exhibited COPD‐like features, including impaired lung function, emphysematous alterations, pulmonary inflammatory cell infiltration, and airway remodeling, accompanied by increased right ventricular systolic pressure, right ventricular hypertrophy, fibrosis, macrophage aggregation, and reduced capillary density.

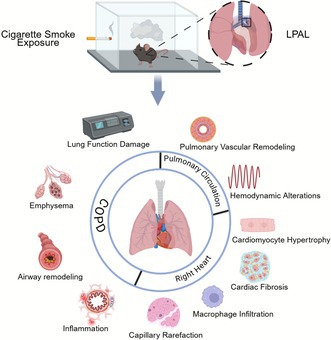

## 1 INTRODUCTION

Cor pulmonale is characterized by right ventricular enlargement and dysfunction due to pulmonary hypertension (PH) caused by various conditions (Fishman, 1976; Rubin, 2018), such as chronic obstructive pulmonary disease (COPD) (Kahnert et al., 2023), pulmonary embolism (Delcroix et al., 2021; Yang et al., 2023), post‐traumatic or postsurgical alterations (Cryer et al., 1990), mechanical ventilation injury (Katira et al., 2017), scleroderma (Haque et al., 2021), and cystic fibrosis (Labombarda et al., 2016), in which COPD is identified as the most common cause (Kahnert et al., 2023; Ruopp & Cockrill, 2022). COPD is a chronic condition distinguished by persistent airflow limitation and chronic airway inflammation that affects approximately 10% of adults worldwide (Kaur et al., 2022). By 2050, the number of COPD patients could reach 592 million (Boers et al., 2023), thereby imposing a substantial economic burden and posing a significant threat to public health due to its high morbidity and mortality rates (Agusti et al., 2023).

In COPD patients, hypoxia‐induced vasoconstriction, hypercapnia, acidosis, inflammation, and reduced pulmonary vascular bed contribute to increased pulmonary vascular resistance and right ventricular afterload, ultimately resulting in the development of right ventricular hypertrophy, fibrosis, and right heart failure (RHF) (MacNee, 1994). A longitudinal study revealed that up to 20% of COPD patients exhibited right heart dysfunction (Andersson et al., 2019), and the coexistence of cor pulmonale in COPD patients was associated with increased hospitalization rates and mortality compared to those without cor pulmonale (Campo et al., 2011; Park et al., 2015). Treatment strategies for patients with COPD‐caused cor pulmonale primarily focused on maintaining pulmonary ventilation through therapeutic aerosols accompanied by using diuretics or digoxin for managing heart failure. However, there are limitations to the effectiveness of these treatment options. For instance, although successful in reducing the pulmonary vascular resistance in patients with pulmonary hypertension, pulmonary vasodilators could not significantly improve symptoms or prognosis in COPD patients (Sakao, 2019). This limitation may be due to the potential of vasodilators to exacerbate hypoxic conditions by causing a ventilation/perfusion mismatch in poorly ventilated lung regions.

Therefore, the development of suitable animal models is essential for investigating effective treatments for COPD‐cor pulmonale. Current understanding of the molecular mechanisms underlying pulmonary vascular lesions in chronic lung diseases has predominantly derived from hypoxia‐based animal models. Nonetheless, these models frequently fail to precisely mimic the intricate alterations in the lungs and right ventricles observed in COPD‐caused cor pulmonale (Boucherat et al., 2022). Chronic cigarette smoke (CS) exposure is a well‐established method for inducing COPD‐like conditions in rodents, leading to pulmonary and systemic inflammation, airway remodeling, emphysema, and compromised lung function (Fricker et al., 2014; Upadhyay et al., 2023). Although these models exhibit some pulmonary vascular change (Wright et al., 2011), they rarely progress to PH or cor pulmonale. To date, no animal model has convincingly demonstrated the intricate interactions between COPD and RHF seen in human patients suffering from COPD‐cor pulmonale. Therefore, we aimed to establish an innovative mouse model that encapsulated the comprehensive range of pathological processes inherent to COPD‐cor pulmonale.

## 2 METHODS

### 2.1 Animal models

An equal number of male and female C57BL/6J mice, aged 6–8 weeks (18–20 g for females and 21–23 g for males), were obtained from the Experimental Animal Center of Guangdong Province. All mice were maintained in a specific pathogen‐free environment with ad libitum access to water and food (GDMLAC007, from Guangdong Medical Laboratory Animal Center, Guangdong, China), adhering to the guidelines of the Animal Care and Use Committee of Guangzhou Medical University. All experimental protocols were reviewed and approved by the Ethics Committee of the Animal Care and Use Committee of Guangzhou Medical University (20230067). The mice were randomly divided into the sham (sham surgery and exposed to air), sham‐CS (sham surgery and exposed to cigarette smoke), Left pulmonary artery ligation (LPAL)‐air (LPAL and exposed to room air), and LPAL‐CS (LPAL and exposed to cigarette smoke). Each group comprised 10 mice, including five male and five female mice.

**FIGURE 1:**
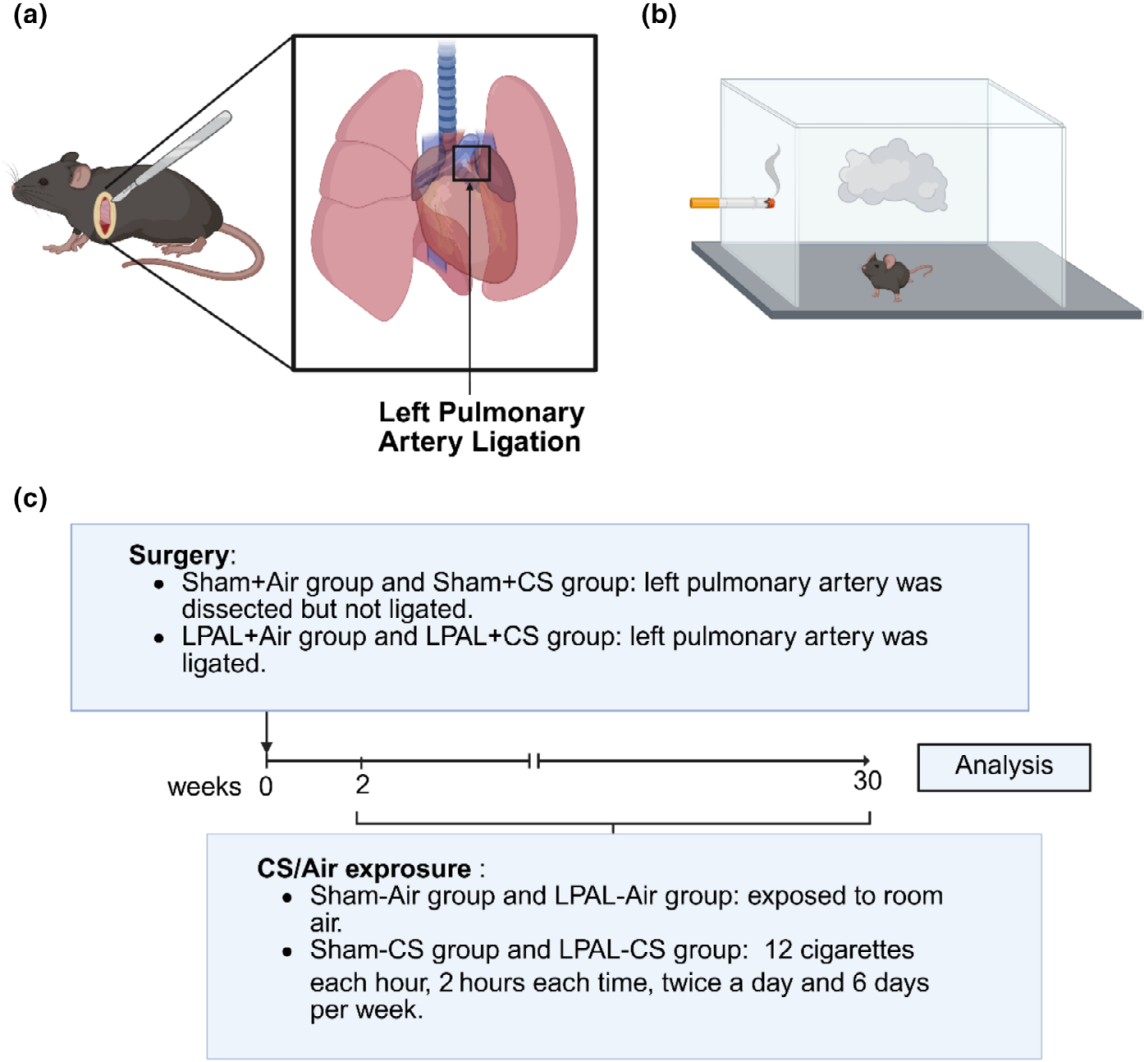
Schematic of surgical procedure and smoke exposure. (a) Surgical procedure: Diagram illustrating the ligation of the left pulmonary artery in mice. (b) Smoke exposure setup: Mice were exposed to cigarette smoke within a whole‐body exposure chamber. (c) Smoke exposure timeline: Cigarette smoke or room air exposure began 2 weeks post‐surgery. Over the 28‐week period, mice in the sham‐CS and LPAL‐CS groups were exposed to cigarette smoke (12 cigarettes per hour, 2 h each time, twice daily, 6 days per week). In contrast, mice in the sham‐air and LPAL‐air groups were exposed to room air in a separate system. (Created with BioRender.com).

A schematic representation of the surgical procedure and smoke exposure setup is shown in Figure 1. The LPAL procedure was performed as previously described (Chen et al., 2022). After being anesthetized by nebulized inhalation of 3% isoflurane (R510‐22‐10, RWD, China), mice were extubated and connected to a ventilator (ROVENT Jr, Kent Scientific, USA) (parameters of the ventilator were set to: normal respiratory mode; respiratory rate: 140–150 breaths/min; tidal volume: 0.12–0.15 mL), while the anesthesia was maintained with 2% isoflurane. In brief, an incision was made in the third intercostal space to access the left lung. The left pulmonary artery was meticulously isolated from the left bronchus using forceps and was ligated with 6–0 nylon sutures under sterile saline‐soaked gauze. Subsequently, the intercostal muscles were sutured with a 4–0 nylon suture, and the skin incision was closed using a tissue adhesive (Cat. No. 1469SB, 3M, St. Paul, MN, USA). In the sham groups, the left pulmonary artery was exposed but not ligated (Huang et al., 2022). During surgical procedures and recovery, animals were maintained on a thermostatically controlled electric heating blanket to preserve core body temperature. Postoperative analgesia was provided for 7 consecutive days via drinking water supplemented with acetaminophen at a concentration of 5 mg/mL (catalog no. 13200001166, Johnson & Johnson Pharmaceutical Ltd., Shanghai, China). Two weeks after the LPAL procedure, mice were exposed to either control air or cigarette smoke of cigarettes (Plum Blossom, Guangdong Tobacco Industry Co. Ltd., Guangzhou, China) (12 cigarettes per hour, 2 h per session, 2 sessions per day, and 6 days per week) (Shu et al., 2017). After 28 weeks of exposure, lung function test and hemodynamic test were performed after anesthesia. The mice were then euthanized with 5% isoflurane, and heart and lung tissues were collected for further examination.

### 2.2 Right ventricular hypertrophy measurements

Briefly, at the conclusion of CS exposure, the mice were anesthetized with 3% isoflurane (R510‐22‐10, RWD, China). The right ventricular systolic pressure (RVSP) was then measured utilizing a 1.2‐F micro‐tip pressure transducer catheter (Millar Instruments, Houston, TX, USA), which was connected to a PowerLab data acquisition system (AD Instruments, Sydney, Australia) (Huang et al., 2022).

### 2.3 Lung function tests

Lung function tests were performed using the Forced Pulmonary Maneuver System (Buxco Research Systems, Wilmington, North Carolina, USA) in accordance with the manufacturer's instructions. Prior to pulmonary function testing, mice were anesthetized by intraperitoneal injection of pentobarbital (MilliporeSigma, Burlington, MA, USA) sodium at a dose of 50 mg/kg. After tracheostomy and intubation, the mice were positioned in the system's chamber. The breathing rate of the anesthetized mice was set at 150 breaths per minute. Various lung function parameters, such as functional residual capacity (FRC), pulmonary dynamic compliance (Cdyn), maximal mid‐expiratory flow (MMEF), forced expiratory volume in 50 ms (FEV50), and forced vital capacity (FVC), were monitored and recorded using a computer scanner.

### 2.4 Hematoxylin and eosin (H&E) and Masson staining

The right upper lobe and left lung of each mouse were slowly perfused with 4% paraformaldehyde for 24 h. The fixed lung tissues were embedded in paraffin and sectioned into 4 μm sections. After dewaxing and rehydration, the sections were stained with hematoxylin (ServiceBio, Wuhan, China) and then stained with eosin (ServiceBio, Wuhan, China). The pathological changes were observed and photographed by using 3D HISTECH Pannoramic MIDI II (3DHISTECH, Hungary). SlideViewer 2.7 (3DHISTECH, Hungary) was used to observe and assess sections.

Masson staining was used to evaluate airway remodeling. Collagen was stained with Sirius Red (GP1033, ServiceBio, China), and cytoplasm was stained with Acid Fuchsin (G2011, ServiceBio, China). The sections were observed, photographed, and assessed as described above. Collagen content was quantified by calculating the ratio of the collagenous surface area (stained blue) to the total surface area based on staining (Shu et al., 2017). The Image J software (NIH, Bethesda, MD, USA) was employed to measure the collagen deposition. The results were expressed as the ratio of collagen surface area to the total surface area.

### 2.5 Morphological analysis

The mean linear intercept (MLI) served as an indicator for measuring the average distance between alveolar walls. It was determined by drawing a line on lung sections and calculating the ratio of the line's length to the total number of alveolar wall intercepts within the line at a magnification of 100×. A total of 36 lines were drawn and measured for each mouse lung (Chen et al., 2009). Lung lesions in each mouse, including interstitial infiltration, perivascular infiltration, bronchus‐associated lymphoid tissue hyperplasia, and peribronchial infiltration, were subjectively graded according to their severity, using a numerical scale from 1 (mild) to 4 (severe). Individual lesion scores were aggregated to derive an overall histopathological score for each animal. All sections were evaluated by pathologists who were blinded to the exposure conditions (Barrett et al., 2002). In each lung section, five small pulmonary vessels with diameters ranging from 30 to 100 μm were selected for measurement. Pulmonary vascular wall thickening was quantified by calculating vascular wall area/total vascular area (Huang et al., 2022).

### 2.6 Right ventricular hypertrophy measurements

The degree of right ventricular hypertrophy in mice was measured by the ratio of heart weight/body weight. The right ventricular free wall thickness (RVWT) was determined by analyzing five evenly spaced (500‐micron intervals) cross‐sections at the mid‐right ventricular (RV) level. RVWT is derived from the average of the midline length of the region occupied by the right ventricular free wall (Zhou et al., 2022). Cardiomyocyte hypertrophy was assessed by measuring the cross‐sectional area of cardiomyocytes. A total of 20 cardiomyocytes were randomly selected from each RV to calculate the mean cross‐sectional area of cardiomyocytes of each mouse (Akazawa et al., 2020).

### 2.7 Immunofluorescence (IF)

IF staining was performed as previously described (Zeng et al., 2023). Briefly, after deparaffinization and antigen retrieval, sections were blocked with 5% Bovine Serum Albumin (BSA, 143064, Biofroxx, Germany) for 1 h at room temperature and then incubated with the primary antibodies (anti‐CD31 (1:200, GB12063, Servicebio, China) and anti‐alpha smooth muscle Actin (α‐SMA) (1:300, GB111364, Servicebio, China)) at 4°C overnight, followed by goat anti‐rabbit (1:400, GB21303, ServiceBio, China) or goat anti‐mouse (1:400, GB25301, ServiceBio, China) at room temperature for 2 h and DAPI (G1012, Servicebio, China) for 10 min. Finally, the sections were washed and mounted using an anti‐fade mounting medium. The images were scanned with 3D HISTECH Pannoramic MIDI II and analyzed using SlideViewer 2.7 software. Image analysis was performed using Image J software (NIH, Bethesda, MD, USA). Right heart capillary density was measured by calculating the capillaries/mm^2^ of tissue area (Zeng et al., 2023). Pulmonary muscularization was assessed in the arteries with a diameter of 50–100 μm. The luminal diameter was the distance from the CD31+ endothelium surface on one side to that on the other side. The area of the smooth muscle layer (α‐SMA+ area) in each muscularized artery was measured. Muscularization severity was expressed as α‐SMA+ area/vessel luminal diameter (Zhou et al., 2022).

### 2.8 Immunohistochemistry (IHC)

Briefly, after deparaffinization and antigen retrieval, the sections were blocked with 3% hydrogen peroxide solution and 5% BSA for 0.5 h at room temperature and then incubated with the primary antibody (Anti‐CD68 (1:200, GB113109, Servicebio, China)) at 4°C overnight, followed by species‐specific horseradish peroxidase (HRP)‐coupled secondary antibodies (1:100, G1214 or G1213, Servicebio, China) at room temperature for 2 h. Finally, HRP staining was conducted with a DAB Chromogenic Kit (G1212, Servicebio, Wuhan, China) according to the manufacturer's instructions. Macrophage numbers were measured by counting the number of CD68‐positive cells per 0.18 mm^2^ of tissue area (Al‐Qazazi et al., 2022).

### 2.9 Statistical analysis

Data was presented as the mean ± standard deviation (SD), derived from three independent experiments. Normality of data distribution was assessed using the Shapiro–Wilk test, and homogeneity of variances was evaluated using the Brown‐Forsythe test. One‐way ANOVA was applied for comparison involving a single independent variable, whereas two‐way ANOVA was chosen for comparison involving two independent variables. Post hoc pairwise comparisons were conducted with Bartlett's test. For nonparametric datasets, the Kruskal–Wallis test was conducted for comparison, complemented by Dunn's test for multiple comparisons. For the analysis of nonparametric data with two dependent variables, pairwise comparisons were conducted using multiple Mann–Whitney tests followed by Holm–Šídák step‐down correction at a family‐wise α of 0.05. The correlation between the two datasets was determined using Spearman's correlation analysis. A *p* value less than 0.05 was considered statistical significance.

## 3 RESULTS

### 3.1 Pulmonary hyperinflation and airflow limitation in mice subjected to LPAL and CS exposure

**FIGURE 2:**
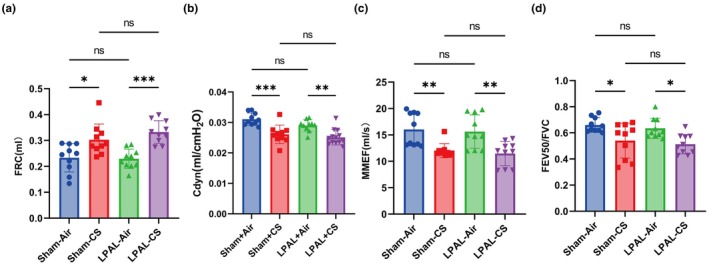
Lung function in smoke‐exposed injured mice. (a) Functional residual capacity (FRC) (*F* (3, 36) = 10.54, *p* < 0.001). (b) Dynamic lung compliance (Cdyn) (*F* (3, 36) = 21.36, *p* < 0.001). (c) Maximal mid‐expiratory flow curve (MMEF) (*F* (3, 36) = 8.462, *p < 0.001*). (d) Forced expiration volume in 50 ms/Forced vital capacity (FEV50/FVC) (*F* (3, 36) = 6.272, *p* = 0.0016). *N* = 10 per group. Data are presented as mean ± standard deviation. Statistical significance: *p < 0.05, **p < 0.01, and ***p < 0.001.

Compared to the air‐exposed groups, mice exposed to CS demonstrated a significantly increased FRC in both sham and LPAL groups (sham‐air vs. sham‐CS, *p* < 0.05; LPAL‐air vs. LPAL‐CS, *p* < 0.001. Figure 2a). Concurrently, compared with the air‐exposed groups, mice in the CS groups had significantly decreased levels of Cdyn (sham‐air vs. sham‐CS, *p* < 0.001; LPAL‐air vs. LPAL‐CS, *p* < 0.001) (Figure 2b), MMEF (sham‐air vs. sham‐CS, *p* < 0.01; LPAL‐air vs. LPAL‐CS, *p* < 0.01) (Figure 2c), and FEV50/FVC (sham‐air vs. sham‐CS, *p* < 0.05; LPAL‐air vs. LPAL‐CS, *p* < 0.05) (Figure 2d). LPAL did not significantly affect these lung function indices, including FRC (sham‐air vs. LPAL‐air, *p* > 0.9999; sham‐CS vs. LPAL‐CS, *p* = 0.7228) (Figure 2a), Cdyn (sham‐air vs. LPAL‐air, *p* = 0.2019; sham‐CS vs. LPAL‐CS, *p* = 0.1834) (Figure 2b), MMEF (sham‐air vs. LPAL‐air, *p* = 0.9996; sham‐CS vs. LPAL‐CS, *p* = 0.9972) (Figure 2c), and FEV50/FVC (sham‐air vs. LPAL‐air, *p* = 0.9884, sham‐CS vs. LPAL‐CS, *p* = 0.9835) (Figure 2d) between sham‐air and LPAL‐air groups, as well as sham‐CS and LPAL‐CS. In addition, age‐matched non‐operated mice exhibited higher FRC and FEV50/FVC than sham‐operated mice, whereas Cdyn and MMEF did not differ significantly, suggesting a modest long‐term effect of sham thoracotomy on selected indices (Figure S1). Taken together, these findings suggest that cigarette smoke is the primary driver of lung functional impairment in this model, while LPAL alone did not significantly alter these lung function parameters.

### 3.2 Lung emphysema, inflammation, and airway remodeling in CS‐induced mice

**FIGURE 3:**
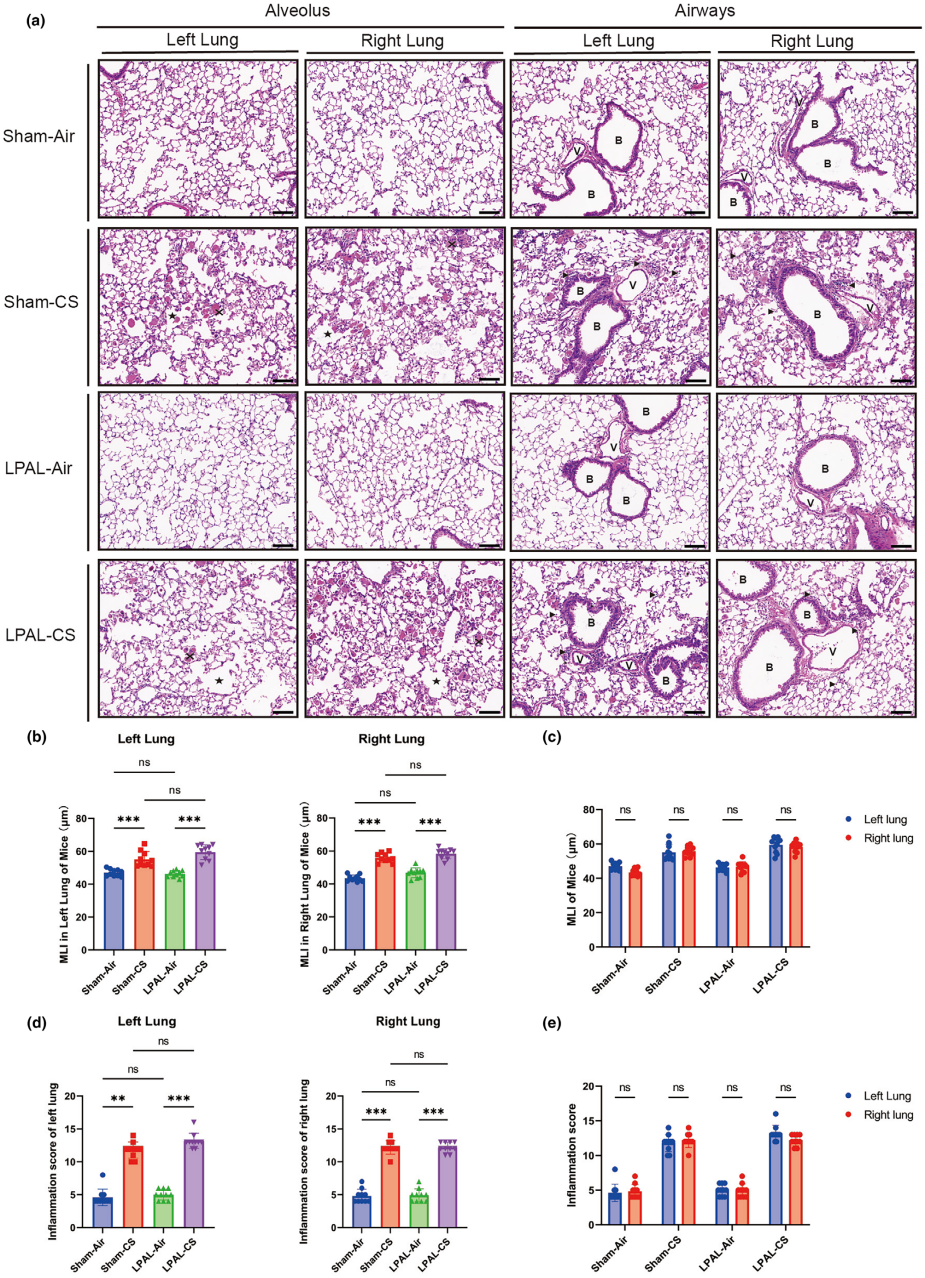
CS exposure induces emphysema and lung inflammation. (a) H&E staining visualized mouse alveoli and airways. Magnification 200× (Scale bar: 100 μm). Dead epithelial cells were marked with ×, Alveolar fusion resulting in enlarged airspaces due to alveolar epithelial cell death was indicated by pentagrams, and inflammatory cells were shown by black arrows. B, bronchus. V, vessel. (b) Mean linear intercept (MLI) of alveoli in the left lung (left panel: *F* (3, 36) = 32.16, *p* < 0.001) and right lung (right panel: *F* (3, 36) = 67.03, *p* < 0.001). (c) Combined MLI of alveoli in both lungs (group *F* (3, 72) = 88.32, *p* < 0.001; lung‐side *F* (1, 72) = 1.664, *p* = 0.2012; interaction *F* (3, 72) = 1.869, *p* = 0.1424). (d) Airway inflammation score in the left lung (left panel: *H* (3, *N* = 40) = 32.24, *p* < 0.001) and right lung (right panel: *H* (3, *N* = 40) = 30.37, *p* < 0.001). (e) Combined airway inflammation score in both lungs (all *p*_adjust_ >0.05). *N* = 10 per group. Data are presented as mean ± standard deviation. Statistical significance: *p < 0.05, **p < 0.01, ***p < 0.001.

Compared to the air‐exposed mice, CS exposure induced the disruption of the alveolar walls and dead epithelial cells, accompanied by the increase in MLI of alveoli (sham‐air vs. sham‐CS, *p* < 0.001; LPAL‐air vs. LPAL‐CS, *p* < 0.001) (Figure 3a,b) and airway inflammation score (Left Lungs: sham‐air vs. sham‐CS, *p* < 0.01; LPAL‐air vs. LPAL‐CS, *p* < 0.001. Right Lungs: sham‐air vs. sham‐CS, *p* < 0.001; LPAL‐air vs. LPAL‐CS, *p* < 0.001) (Figure 3a,d) in both left and right lungs. Notably, the presence of LPAL did not influence emphysema or airway inflammation compared with the sham groups (Figure 3c,e).

**FIGURE 4:**
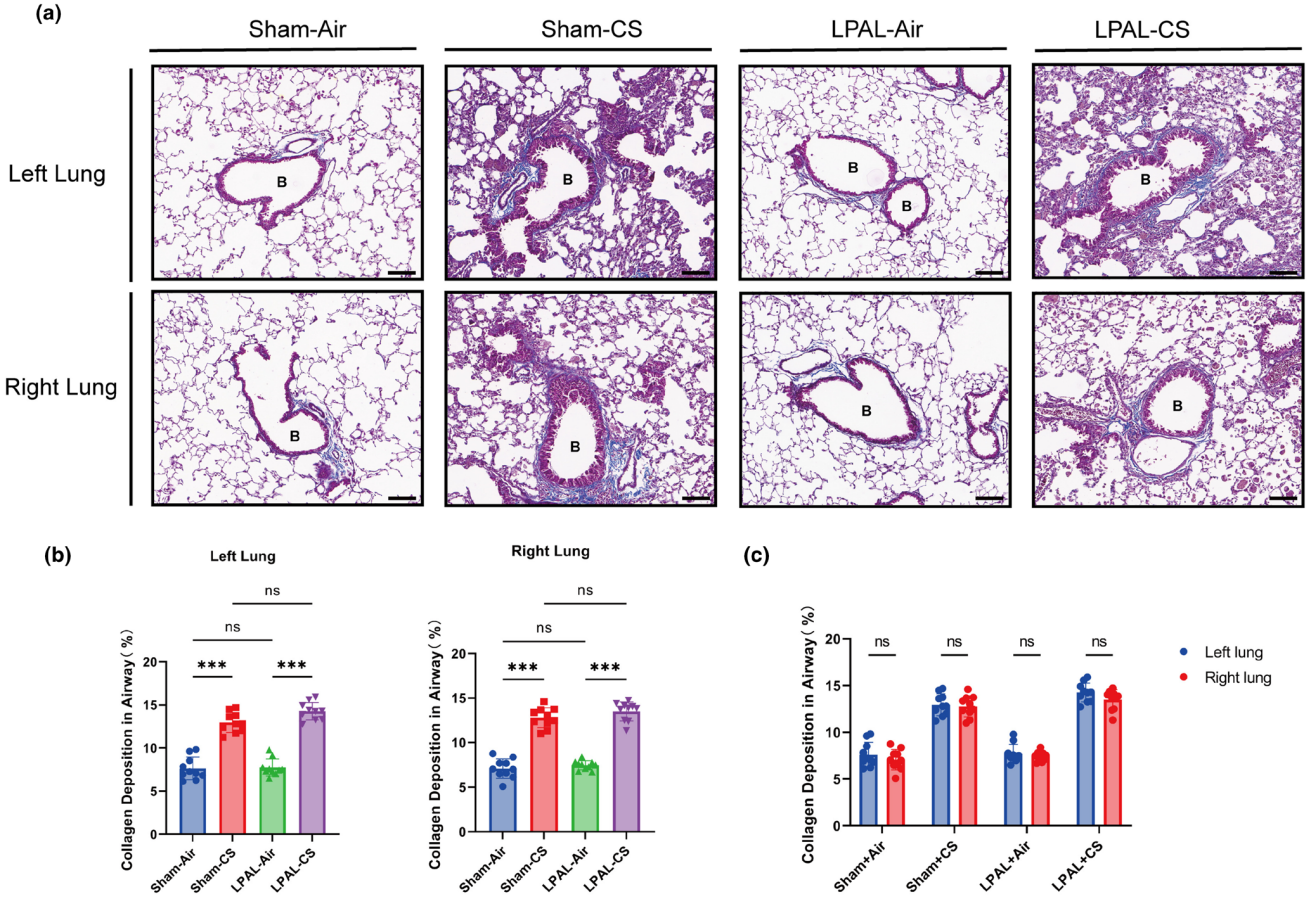
Bronchial remodeling. (a) Masson staining highlighted bronchial remodeling in the lungs, magnification 200× (Scale bar: 100 μm). B, bronchus. (b) Proportion of collagen deposits in airways of the left lung (left panel: *F* (3, 36) = 94.09, *p* < 0.001) and right lung (right panel: *F* (3, 36) = 119.9, *p* < 0.001). (c) Combined proportion of collagen deposits in the airways of both lungs (group *F* (3, 72) = 210.4, *p* < 0.001; lung‐side *F* (1, 72) = 35.36, *p* = 0.641; interaction *F* (3, 72) = 0.3255, *p* = 0.8069). *N* = 10 per group. Data are presented as mean ± standard deviation. Statistical significance: *p < 0.05, **p < 0.01, ***p < 0.001.

Airway collagen deposition, a key feature of airway remodeling, was significantly increased in both the left and right lungs of CS‐exposed mice compared to air‐exposed mice (sham‐air vs. sham‐CS, *p* < 0.001; LPAL‐air vs. LPAL‐CS, *p* < 0.001) (Figure 4a,b). Similarly, LPAL did not further increase airway collagen deposition compared with the sham groups (*p* > 0.05) (Figure 4a,c).

In summary, the findings indicate that CS exposure in this study reliably induced key COPD‐like lung phenotypes, including emphysema, inflammatory infiltration, and airway collagen remodeling. Thereby establishing a robust pulmonary disease background for subsequent evaluation of pulmonary vascular changes and right ventricular remodeling in the LPAL‐CS right heart failure model.

### 3.3 Vascular remodeling and elevated right ventricular systolic pressure

**FIGURE 5:**
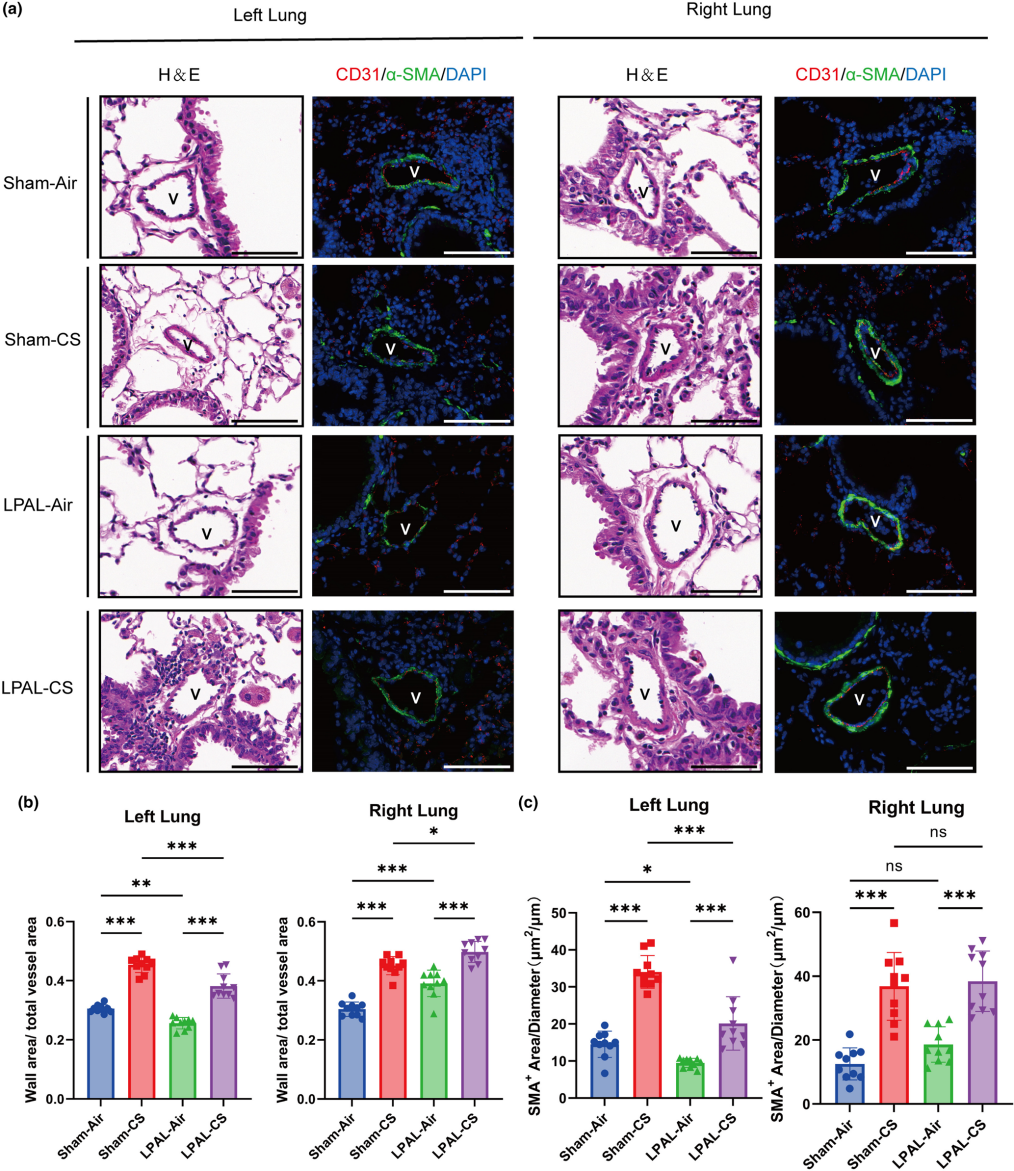
Pulmonary vascular remodeling. (a) H&E staining visualized small blood vessels, and immunofluorescence staining for CD31 (red) and α‐SMA (green) was performed in mice's lungs, magnification 630× (Scale bar: 100 μm). (b) Wall area/total vessel area ratio in the left lung (left panel: *F* (3, 36) = 102.2, *p* < 0.001) and right lung (right panel: *F* (3, 36) = 59.06, *p* < 0.001). (c) Pulmonary arterial remodeling was quantified by calculating the α‐SMA‐positive area/vessel diameter ratio in the left lung (left panel: *F* (3, 36) = 57.22, *p* < 0.001) and right lung (right panel: *F* (3, 36) = 26.21, *p* < 0.001). *N* = 10 per group. Data are presented as mean ± standard deviation. Statistical significance: *p < 0.05, **p < 0.01, ***p < 0.001.

**FIGURE 6:**
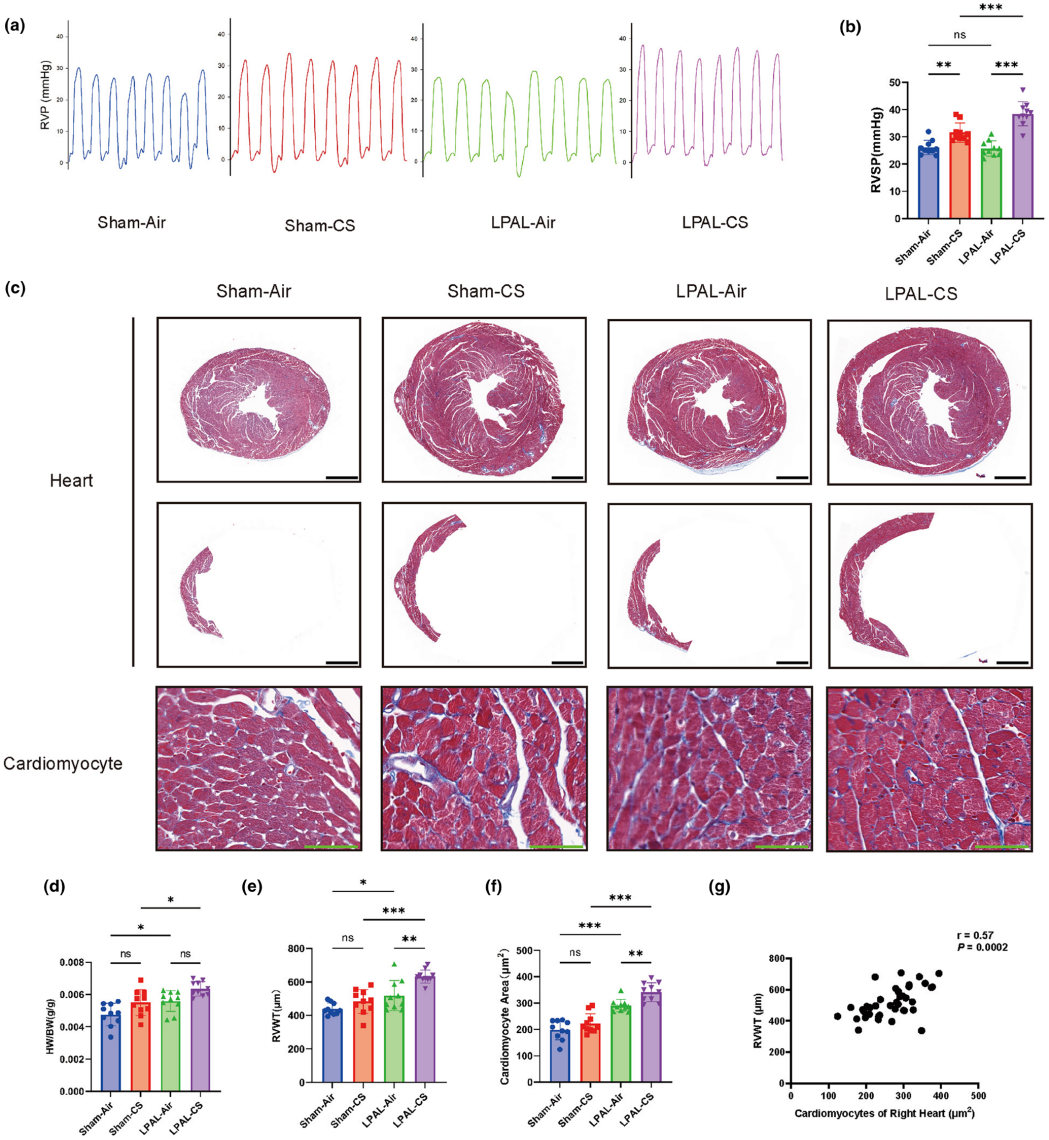
Hemodynamic changes and right heart thickening. (a) Right ventricular (RV) pressure waveform morphology in mice. (b) Right ventricular systolic pressure (RVSP) measurements in mice (*F* (3, 36) = 30.72, *p* < 0.001). (c) Masson staining of mouse hearts revealed right heart hypertrophy. Cross‐sections of mouse hearts at the mid‐RV plane (top) and a highlighted view of the RV (middle) are shown, magnification 30× (Scale bar: 1 mm). Additionally, Masson staining of cardiomyocytes in the right heart is presented (bottom), magnification 630× (Scale bar: 100 μm). (d) Ratio of heart weight to body weight (HW/BW) in mice (*F* (3, 36) = 9.857, *p* < 0.001). (e) Right ventricular free wall thickness (RVWT) (*F* (3, 36) = 17.49, *p* < 0.001). (f) Quantification of right ventricular cardiomyocyte area (*F* (3, 36) = 37.25, *p* < 0.001). (g) Correlation analysis between RVWT and right heart cardiomyocyte area. *N* = 10 per group. Data are presented as mean ± standard deviation. Statistical significance: *p < 0.05, **p < 0.01, ***p < 0.001.

Vessel wall thickness in the right lung arteries significantly increases after LPAL compared with the sham group (sham‐air vs. LPAL‐air, *p* < 0.001; sham‐CS vs. LPAL‐CS, *p* < 0.05). In contrast, the left lung arteries of mice that underwent LPAL exhibited thinner vascular walls compared to the sham groups (sham‐air vs. LPAL‐air, *p* < 0.01, sham‐CS vs. LPAL‐CS, *p* < 0.001) (Figure 5a,b). Moreover, a significant reduction in vascular muscularization was observed in the left lung of mice that underwent LPAL (sham‐air vs. LPAL‐air, *p* < 0.05; sham‐CS vs. LPAL‐CS, *p* < 0.001). However, no significant increases in vascular muscularization were observed in the right lung of mice that underwent LPAL (sham‐air vs. LPAL‐air, *p* = 0.4602, sham‐CS vs. LPAL‐CS, *p* = 0.9983) (Figure 5a,c). The blood vessels in the left and right lungs were both significantly thickened in mice after CS exposure, regardless of pulmonary vascular ligation (sham‐air vs. LPAL‐air, *p* < 0.001; sham‐CS vs. LPAL‐CS, *p* < 0.001) (Figure 5a,b). Additionally, the severity of vascular myelination was increased after CS exposure in comparison with the air exposure (sham‐air vs. LPAL‐air, *p* < 0.001; sham‐CS vs. LPAL‐CS, *p* < 0.001) (Figure 5a,c). Furthermore, both CS exposure and LPAL alone elevated RVSP (sham‐air vs. sham‐CS, *p* < 0.01; sham‐air vs. LPAL‐air, *p* < 0.05), with a synergistic effect observed when CS exposure combined with LPAL (sham‐CS vs. LPAL‐CS, *p* < 0.01; LPAL‐air vs. LPAL‐CS, *p* < 0.001) (Figure 6a,b). The findings reveal that exposure to both LPAL and CS was a contributing factor to the observed increased pulmonary vascular remodeling and RVSP.

### 3.4 Right heart hypertrophy induced by LPAL combined with CS

The severity of right heart hypertrophy was evaluated using the HW/BW ratio and RVWT. The HW/BW ratios remained unchanged when CS exposure was compared to air exposure (sham‐air vs. sham‐CS, *p* = 0.0895). However, when LPAL was combined with CS exposure, a significant increase in the HW/BW ratio was observed compared to mice exposed to CS exposure alone (sham‐CS vs. LPAL‐CS, *p* < 0.05) (Figure 6d).

CS exposure tended to increase the RVWT, without statistical significance (sham‐air vs. sham‐CS, *p* = 0.5194). In contrast, right ventricular hypertrophy was significantly greater in the LPAL‐CS group compared to both the sham‐CS and LPAL‐air groups (sham‐CS vs. LPAL‐CS, *p* < 0.001; LPAL‐air vs. LPAL‐CS, *p* < 0.01, Figure 6c,e). There was no significant difference in the area of right heart cardiomyocytes between the sham‐CS and sham‐air groups (*p* = 0.3783). However, LPAL alone was sufficient to induce right heart cardiomyocyte hypertrophy (sham‐air vs. LPAL‐air, *p* < 0.001), and the LPAL‐CS group exhibited even greater hypertrophy compared to the LPAL‐air group (LPAL‐air vs. LPAL‐CS, *p* < 0.01) (Figure 6c,f). Additionally, a positive correlation was found between the area of right heart cardiomyocytes and RVWT across all groups (Figure 6g). Overall, these findings suggest that the combined administration of LPAL and CS led to increased right heart hypertrophy in mice, followed by RHF.

### 3.5 Right ventricle fibrosis, macrophage infiltration, and capillary rarefaction induced by CS exposure and LPAL

**FIGURE 7:**
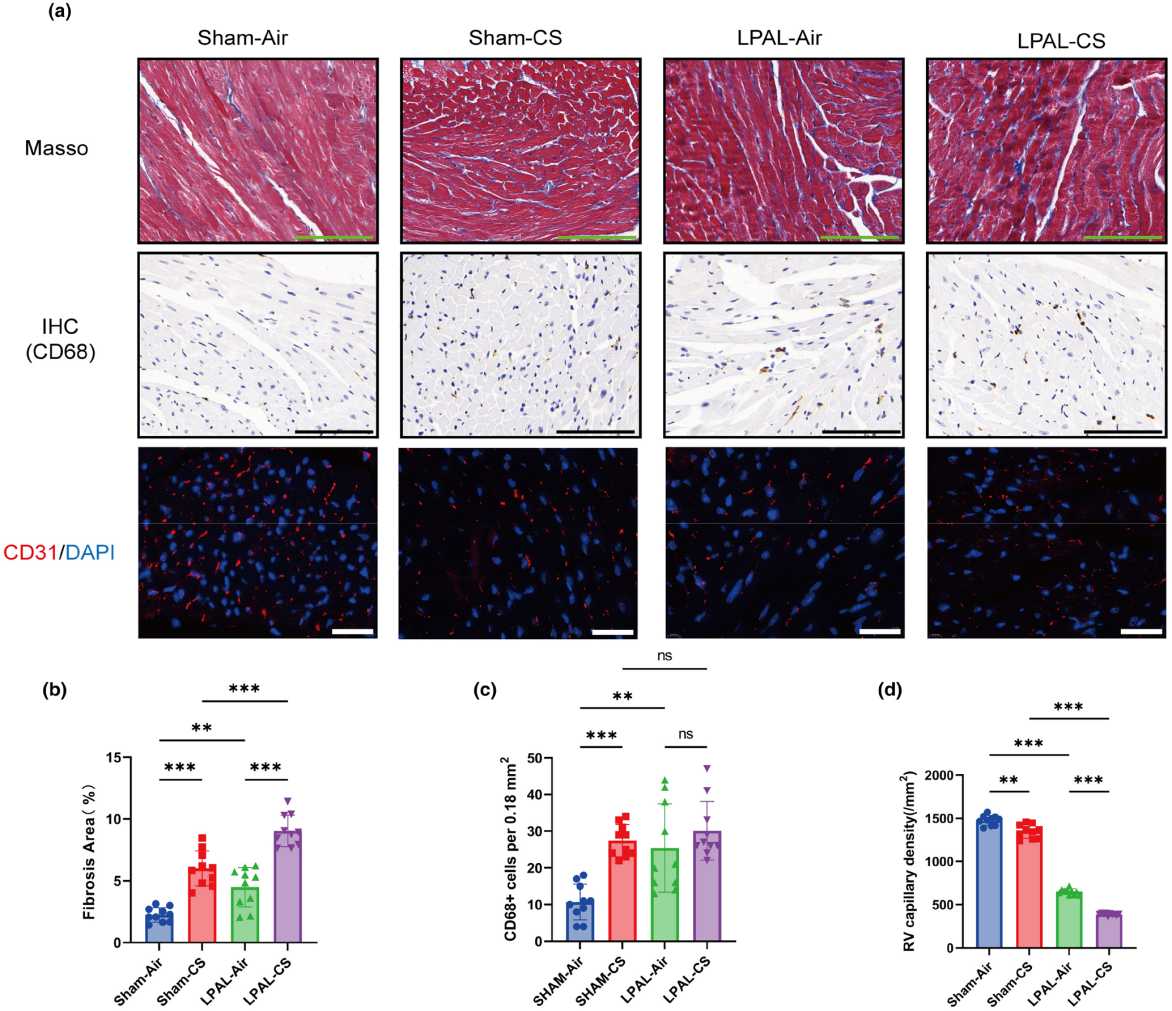
Right heart fibrosis and cardiomyocyte hypertrophy. (a) Masson staining revealed the presence of right heart fibrosis in mice (top panel, magnification 630×, Scale bar: 100 μm). Immunohistochemical analysis for CD68 (brown) demonstrated the distribution of macrophages within the right heart (middle panel, magnification 630×, Scale bar: 100 μm). Additionally, immunofluorescence staining for CD31 (red) was performed on mouse hearts (bottom panel, magnification 1000×, Scale bar: 30 μm). (b) The percentage of collagen deposition in the right heart of mice was quantified (*F* (3, 36) = 50.33, *p* < 0.001). (c) The number of CD68+ cells per 0.18 mm^2^ was determined (*F* (3, 36) = 11.96, *p* < 0.001). (d) Capillary density in the right ventricle was assessed (*H* (3, *N* = 40) = 35.39, *p* < 0.001). *N* = 10 per group. Data are presented as mean ± standard deviation. Statistical significance: *p < 0.05, **p < 0.01, ***p < 0.001.

Both CS exposure and LPAL independently and significantly increased right ventricular fibrosis (sham‐air vs. sham‐CS, *p* < 0.001; sham‐air vs. LPAL‐air: *p* < 0.01). Moreover, the combination of CS exposure and LPAL induced a more pronounced increase in fibrosis compared to either treatment alone (*p* < 0.001) (Figure 7a,b).

Macrophage infiltration plays a crucial role in the development of COPD and heart dysfunction (Al‐Qazazi et al., 2022; Kapellos et al., 2018). Either CS exposure or LPAL alone significantly increased macrophage aggregation in the right ventricle compared to the control group (*p* < 0.01). Interestingly, the combination of CS exposure with LPAL did not lead to a further increase in macrophage infiltration in the right ventricular free wall compared to the control group (*p* > 0.05) (Figure 7a,c).

The adaptive remodeling process of the RV frequently involves a reduction in capillary density (Frump et al., 2018). By employing CD31 immunofluorescence to visualize capillaries, we noted that both CS exposure and LPAL independently led to a decrease in RV capillary density (sham‐air vs. sham‐CS, *p* < 0.01; sham‐air vs. LPAL‐air, *p* < 0.001). Moreover, when CS exposure and LPAL were applied concurrently, a significantly more severe reduction in capillary density was observed compared to either treatment alone (*p* < 0.001) (Figure 7a,d). Together, we found that CS exposure combined with LPAL induces right ventricular fibrosis, macrophage infiltration, and capillary rarefaction, reflecting cor pulmonale with RHF‐relevant remodeling.

## 4 DISCUSSION

COPD patients are prone to developing PH, a condition that can increase the afterload on the RV, potentially leading to RHF (Hoeper et al., 2016). Although CS exposure alone is an effective method for inducing COPD in mice, it typically results in only mild pulmonary hypertension and slight right ventricular dysfunction. The severity of cor pulmonale is associated with hypoxemia, hypercarbia, and the extent of airway obstruction. It has been reported that thrombosis and reduction in pulmonary vascular beds are prevalent in patients with COPD‐cor pulmonale (Armentaro et al., 2023; Wang et al., 1998). These factors contribute to increased pulmonary vascular resistance and elevated pulmonary artery pressure. To mimic these vascular alterations, LPAL is introduced into the COPD model in mice (Yun et al., 2015), which effectively induces key clinical features associated with COPD‐cor pulmonale, including pulmonary emphysema, pulmonary vascular remodeling, RV dysfunction, and fibrosis.

COPD is frequently associated with mild‐to‐moderate PH (Andersen et al., 2012; Zhang et al., 2022). The mild elevation of pulmonary artery pressure observed in mice subjected to LPAL combined with CS exposure aligns with the clinical manifestations seen in patients with COPD‐related cor pulmonale (Hilde et al., 2013). These findings suggest that hemodynamic modifications arise from both the direct effects of LPAL and the synergistic relationship between CS and LPAL.

Cigarette smoke contains a complex array of substances, including nicotine, tar, carbon monoxide, and other toxic compounds. Oakes et al. have demonstrated that exposure to nicotine smoke for 8 weeks can lead to right ventricular hypertrophy in mice, characterized by an increase in right ventricular weight and a tendency for right ventricular internal diameter expansion, without causing significant structural or functional changes in the left ventricle (Oakes et al., 2020). This suggests that cigarette smoke may have a selective impact on the RV. Furthermore, inflammation induced by cigarette smoke may directly influence changes in the pulmonary arteries and contribute to right ventricular dysfunction. Prolonged exposure to cigarette smoke, such as for 32 weeks, has been shown to increase heart rate in mice, along with mitochondrial swelling, disruption of the mitochondrial membrane, partial lysis of myogenic fibers in cardiomyocytes, and increased cardiac collagen deposition (Dai et al., 2024). These changes underscore the potential direct and indirect effects of cigarette smoke on the cardiovascular system, particularly the right side of the heart.

The synergistic effects of CS exposure and LPAL on the development of PH and cor pulmonale highlight the multifactorial nature of COPD‐related cor pulmonale. This finding is critical as it emphasizes the complexity of the disease process and underscores the necessity of a comprehensive understanding of the interactions among various factors that drive the progression of COPD to pulmonary heart disease.

LPAL leads to impaired blood supply to the left pulmonary artery. Although there are no significant differences in airway inflammation and remodeling between the left and right lungs under CS exposure, the uneven blood flow into the lungs affects the remodeling of small pulmonary arteries. Mice subjected to LPAL exhibit a notable thickening of the right lung wall compared to the left lung, alongside atrophy of the vascular smooth muscle in the left pulmonary artery. After ligation of the left pulmonary artery, the left lung enters a state of ischemia, while increased blood flow to the right lung results in compensatory hypertrophy of the right small pulmonary artery. While some studies have suggested that ischemia might lead to alveolar destruction and emphysema (DeVries et al., 1979), results of this study indicated that the severity of emphysema did not differ between the left and right lungs in LPAL‐induced mice. This phenomenon could be ascribed to the novel supply that originates principally from the parietal pleura, which is supplied by the intercostal arteries, thereby attenuating left lung ischemia (Mitzner et al., 2000). While some researchers have reported bilateral lung injury occurring 48 h after LPAL (Marongiu et al., 2021), in this study, there were no differences in lung inflammation and lung functions between the LPAL‐air group and the sham‐air group. This may have been due to the extended modeling time in this study. Left pulmonary blood flow increased 5–6 days after LPAL and reached a plateau at the 2nd–3rd weeks (Mitzner et al., 2000). This allowed the initial lung injury to subside.

There are also some limitations to this study. It should be acknowledged that while the LPAL‐CS model provides significant insights, it may not fully replicate the complex interactions between inflammatory processes, vascular changes, and parenchymal alterations that occur over time in COPD patients. Furthermore, this model might not encompass the effects of other comorbidities prevalent in COPD patients, which could play a role in the progression of PH and cor pulmonale.

## 5 CONCLUSION

The LPAL‐CS mouse model developed in this study provides a robust platform for investigating cor pulmonale in the context of COPD. This development is particularly significant as it highlights the complexity of the disease process and the necessity for a comprehensive understanding of the various factors influencing the progression of cor pulmonale in COPD patients.

## AUTHOR CONTRIBUTIONS

TW, XC, and NW conceived and designed the study, devised and supervised the project. QB, YH, and ZM finished the animal experiments. XW and GC wrote the draft of the manuscript. XW, GC, XW, and KZ finished the data analysis. XW and SZ prepared figures. XW, GC, KZ, and TW finished the revision of the manuscript. All authors reviewed the manuscript and approved the final draft for publication and agree to be accountable for all aspects of the work in ensuring that questions related to the accuracy or integrity of any part of the work are appropriately investigated and resolved.

## FUNDING INFORMATION

This work was supported by grants from the National Natural Science Foundation of China (82470030, 82070038, 82241024, 81970046, and 82270053) and Guangdong Outstanding Young Scientist Funding (2021B1515020006).

## CONFLICT OF INTEREST STATEMENT

The authors declare that they have no competing interests.

## ETHICS STATEMENT

The animals were maintained in a specific pathogen‐free environment, adhering to the guidelines set by the Animal Care and Use Committee of Guangzhou Medical University. All experimental protocols were reviewed and approved by this committee (No. 20230067). Furthermore, all testing procedures were conducted following the standards and regulations endorsed by the same committee.

## CONSENT

Not applicable.

## Supporting information


Figure S1.

## Data Availability

The data that support the findings of this study are available from the corresponding author upon reasonable request.
