# Supplementary material for: Simulating Cor pulmonale in chronic obstructive pulmonary disease via cigarette smoke exposure and left pulmonary artery ligation in mice
Source: Physiol Rep. 2026 Jan 9;14(1):e70727. doi: 10.14814/phy2.70727 (PMC12789187; doi:10.14814/phy2.70727)
Supplement: Supplementary file 1 — Figure S1. [file PHY2-14-e70727-s001.docx]

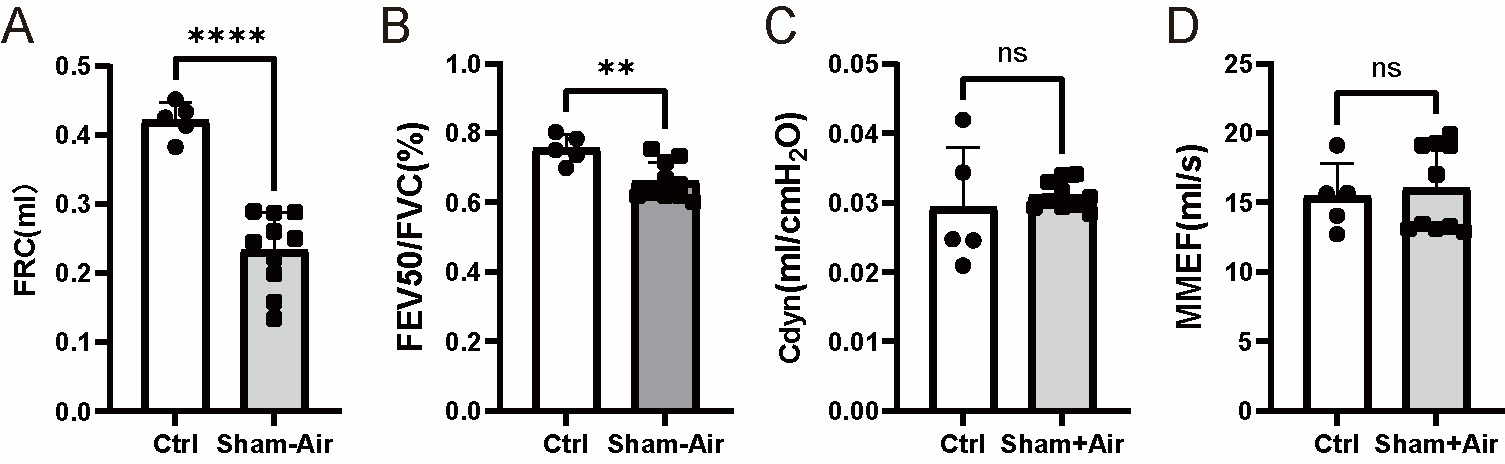


Figure S1 Lung Function in non-operated mice and sham-operated mice. The Sham group exhibited significantly impaired lung function compared to the non-operated group, as evidenced by reduced (A) functional residual capacity (FRC) and (B) forced expiratory volume in 100 milliseconds/forced vital capacity (FEV50/FVC) ratio. And Sham group showed no significant differences of (C) dynamic compliance (Cdyn) and (D) Maximal Mid-Expiratory Flow Curve (MMEF). N=5-10 per group. Data are presented as mean ± standard deviation. Statistical significance: **p<0.01, and ****p<0.0001.
